# Ventricular arrhythmias originating from the basal septum of the ventricle: Clinical and electrophysiological characteristics and a systematic ablation approach

**DOI:** 10.3389/fcvm.2022.879381

**Published:** 2022-11-21

**Authors:** Linsheng Shi, Cheng Wang, Hongwu Chen, Gang Yang, Kai Gu, Mingfang Li, Ming Chu, Hailei Liu, Zidun Wang, Weizhu Ju, Minglong Chen

**Affiliations:** ^1^Department of Cardiology, The Second Affiliated Hospital of Nantong University, Nantong, China; ^2^Nantong School of Clinical Medicine, Kangda College of Nanjing Medical University, Nanjing, China; ^3^Department of Cardiology, The First Affiliated Hospital of Nanjing Medical University, Nanjing, China

**Keywords:** basal septum, ventricular arrhythmias, catheter ablation, anatomy, electroanatomic mapping

## Abstract

**Background:**

There is a paucity of data about VAs clustered at the vicinity of the basal septum of the ventricle. We aimed to report and characterize the clinical and electrophysiological features of basal septum VAs and explore the systematic ablation approach.

**Methods:**

A consecutive series of 51 patients who had their VAs successfully ablated at the basal septum of the ventricle was enrolled in this study. The basal septum was defined as the area 2 cm away from the septal annulus, the upper boundary was the site of the left or right His-Purkinje system, and the lower boundary was the borderline that separated away from the septum. RFCA was performed based on detailed activation mapping or pace mapping. Patients who underwent VA ablation from other areas of the tricuspid annulus (TA) and mitral annulus (MA) during the same period were enrolled as the control group.

**Results:**

The patients with basal septum VAs were significantly older (*p* < 0.01) and had more comorbidities (hypertension and coronary artery disease) (*p* < 0.01). Meanwhile, the precordial R wave transition was significantly different in right side, left side and intramural foci group (*p* < 0.001). Acute procedural success was achieved in 44 patients (86.3%) in the study group and in 63 patients (95.5%) in the control group. After a median of 12 (6–36) months of follow-up, compared with VA recurrence in the control group (2 cases), 11 patients with basal septum VAs had recurrences (*p* = 0.002), while a delayed cure was observed in 3 in intramural foci group.

**Conclusion:**

Based on the unique anatomical and electrophysiological characteristics, a systematic approach for VAs originating from the basal septal area is warranted. Moreover, the follow-up data seemed to show a relative high recurrence rate for basal septal VAs during a period of time.

## Introduction

Ventricular arrhythmias (VAs) in patients without structural heart disease have a predilection for specific anatomic structures. To date, numerous reports have described the ablation of VAs originating from a certain anatomical structure, such as the papillary muscles, the His-Purkinje system (His), the areas near the ventricular outflow tracts, and the annulus (1–5). This clustering feature makes it possible to estimate the exact locations before intracardiac mapping, consequently allowing preprocedure planning and potentially facilitating mapping and ablation. Till now, a relatively large data of VAs clustered at the vicinity of the basal septum of the ventricle are still limited (6–8). In the present study, we report a group of VAs clustered in the vicinity of the basal septum. Additionally, we characterize the clinical and electrophysiological features of basal septum VAs, as well as the elaboration of the systematic ablation approach.

## Materials and methods

### Patient

From February 2012 to April 2021, a consecutive series of 51 patients who had their VAs successfully ablated at the basal septal area was enrolled in this study. These patients were selected from a pool of 1,065 VAs patients referred to the First Affiliated Hospital of Nanjing Medical University. Patients ablated from other areas of the tricuspid annulus (TA) and mitral annulus (MA) during the same period were enrolled as controls. All patients provided written informed consent before the procedure, and the study protocol was approved by the Institutional Review Board of the First Affiliated Hospital of Nanjing Medical University.

### Electrophysiological study, mapping and ablation of the ventricular arrhythmias

All procedures were performed under conscious sedation and local anesthesia. All antiarrhythmic medications were ceased for at least 5 half-lives before the procedure. If needed, a 6F quadripolar and a decapolar catheter were advanced into the high right atrium or right ventricle and coronary sinus, respectively. The isoproterenol challenge was performed to provoke clinical VAs in the absence of VAs during the session.

Three-dimensional mapping was performed using the CARTO (Biosense-Webster Inc., Diamond Bar, CA, USA) or EnSite Velocity (Endocardial Solutions, Inc., Minneapolis, MN, USA) systems. The right ventricle and left ventricle models were reconstructed using the roving catheter via the right femoral vein or right femoral artery with a retrograde aortic approach or a trans-septal approach. Detailed activation mapping was performed in the area of interest to identify the earliest activation site. Remapping at the bilateral side of the basal septum was performed in 18 patients whose initial ablation failed. Additionally, in some cases, pace mapping was performed with bipolar pacing in the lowest possible capture threshold.

Radiofrequency energy delivery was applied to the earliest activation sites using an irrigated ablation catheter (Navistar Thermocool, Biosense Webster, Diamond Bar, CA, USA or IBI Coolflex, St. Jude Medical, St. Paul, MN, USA). The maximum ablation power was adjusted between 20 and 50 W, depending on the proximity to the conduction system at the discretion of the operator. The temperature limit was set at 43°C, and the irrigation rate was 17–0 ml/min. Generally, the site where energy application was attempted would be regarded as an excellent target if the VAs disappeared in 10 s. Consequently, the energy delivery would be performed up to 90 s. If the ablation failed, a remapping was performed to identify the earliest activation site, and then another ablation attempt was made. In some situations, anatomical ablation on both sides of the septum was performed. The endpoint of the procedure was defined as the disappearance of spontaneous clinical arrhythmias and the lack of inducibility of the VA using the same drug provocation protocol as before ablation.

Ventricles were divided into 3 parts equally from the apex to base and the most basal part of the ventricle septum was considered as the basal septum. Moreover, the basal septum was defined as the area 2 cm away from the septal annulus, the upper boundary was the site of the left or right His, and the lower boundary was the borderline that separated from the septum. Those VAs with their earliest activation superior to the His point were not enrolled; as such, no patient was found to need ablation at the right coronary cusp.

Two approaches, that is above-valve and under-valve approach, were used for the ablation of right septum. For the above-valve approach, a long-fixed sheath was used to stabilize the ablation catheter and the catheter was advanced to the above-valve region directly. For the under-valve approach, the catheter was made a big curve and pulled back to reach the TA from the ventricular side.

### Follow-up

After the procedure, patients were sent back to the cardiovascular ward and monitored for at least 12 h. No anti-arrhythmia drugs were used. After discharge, patients were asked to perform pulse checks and undergo 24-h Holter monitoring 3, 6, and 12 months after ablation, with 3- and 6-month intervals post-procedure thereafter to reassess any recurrence of symptoms. During the follow-up, a decrease in PVC burden of over 90% or no recurrent VT was defined as no recurrence. A late cure was defined as the disappearance of the VAs as assessed by Holter after discharge during follow-up.

### Statistical analysis

Continuous variables were expressed as the means ± standard deviations (normally distributed variables) or medians (25th, 75th percentiles). The normality of the distribution was assessed with the Shapiro–Wilk test. Comparisons between groups were performed with an unpaired Student’s *t*-test or non-parametric test. Three group data were analyzed with one-way ANOVA. Categorical variables were expressed as numbers and percentages. The chi-square test or Fisher’s exact test was employed to compare the categorical data. All tests were 2-sided, and a *p* value <0.05 was considered to indicate statistical significance. Statistical analyses were performed using SPSS 22.0.

## Results

### Patient characteristics

A total of 51 studied patients (study group) and 66 control patients (control group) were enrolled in the study. The baseline clinical characteristics are provided in Table 1. No significant differences were noted in sex, left ventricular ejection fraction (LVEF), or left ventricular end diastolic dimension (LVEDD) between the two groups (all *p* > 0.05). However, the patients in the study group were significantly older (59.6 ± 15.5 vs. 34.9 ± 17.3, *p* < 0.01) and had more comorbidities (hypertension and coronary artery disease) than those in the control group (*p* < 0.001). Before the ablation procedure, the patients in the two groups had taken 0.9 ± 0.7 types of medications, including β-blockers, propafenone, mexiletine, amiodarone, and traditional Chinese drugs, with no benefit. One patient in the study group was involved in an electrophysiological study and ablation procedure in another hospital.

**TABLE 1:** Comparisons of clinical and electrophysiological characteristics of VAs in basal septum (studied group) and other area of tricuspid annulus and mitral annulus (control group).

Variables	Study group (51)	Control group (66)	*P*-value
Age (years)	59.6 ± 15.5	34.9 ± 17.3	0.000
Gender (male)	31	37	0.233
**Co-morbidity**			
Hypertension	20	11	0.001
Coronary artery disease	15	0	0.000
Diabetes	4	2	0.177
**Echocardiogram**			
LVEF (%)	62.1 ± 5.3	63.7 ± 3.6	0.051
LVEDD (mm)	48.7 ± 3.9	48.70 ± 4.4	0.402
Burden	24,108.2 ± 12,126.8	23,384.1 ± 11,770.3	0.804
**Medications**			
Beta blockers	25	28	0.072
Class I agents	8	21	0.309
Traditional Chinese drug	13	4	0.019
Total success	36 (70.6%)	61 (90.9%)	0.001
Recurrence during follow up	11 (21.6%)	2 (3.0%)	0.002

Values are expressed as mean ± SD or number (percentage). LVEF, left ventricular ejection fraction; LVEDD, left ventricular end diastolic dimension.

### ECG characteristics

As a result, tall R or RR’ pattern waves were all found in leads I and aVL. In all but 5 patients, the surface ECG demonstrated an initial q or QS wave at lead V1, and the other 5 patients demonstrated an R or Rs pattern. There was a significant difference between Group 1, Group 2 and Group 3 in terms of the morphological features in the precordial lead (Table 2) (*p* = 0.001). The V1 lead showed a positive wave (qR type) in only one patient in Group 3, while in Group 2, a positive wave was found in 7/16 patients (QR, R, RS, or qR type). At the V1 lead, a QS type was found in 34/35 patients in Group 1, while it was found in 9/14 patients in Group 2 and 8/9 patients in Group 3 (*p* = 0.001). In Group 1, the precordial R wave transition occurred at or beyond V3 in 18 patients (64.3%); however, 2 patients in Group 2 exhibited the precordial R wave transition at or beyond V3 (14.3%), and 4 patients in Group 3 (*p* < 0.001).

**TABLE 2:** Comparisons of ECG and electrophysiological characteristics of VAs in basal septum between the right side (Group 1), left side (Group 2) and intramural foci (Group 3).

Variables	Group 1 (28)	Group 2 (14)	Group 3 (9)	*P*-value
**ECG characteristics**				
QRS type in V1				0.001
QS	28	7	8	
QR	0	1	0	
R	0	4	0	
RS	0	1	0	
qR	0	1	1	
Early QRS transition				0.000
(V1)	0	6	1	
(V2)	10	6	4	
(V3)	13	2	2	
(V4)	4	0	1	
(V5)	1	0	1	
Preceding time of the target	26.6 ± 7.8	23.5 ± 6.4	23.4 ± 5.7	0.313
Small “a” at the target	26	13	6	0.353
Total success	20 (71.4%)	11 (78.6%)	5 (55.6%)	0.499

Values are expressed as mean ± SD or number (percentage).

### Mapping and acute ablation outcome

First, an under-valve approach was used to locate the earliest activation of the VAs. In the study group, all of the earliest activations were located at the basal septum (40 patients on the right side and 11 patients on the left side), with a mean preceding time compared with the onset of the QRS wave of 25.4 ± 7.6 ms. A small, far-field “a” signal was recorded at the earliest activation in 45 patients.

**FIGURE 1:**
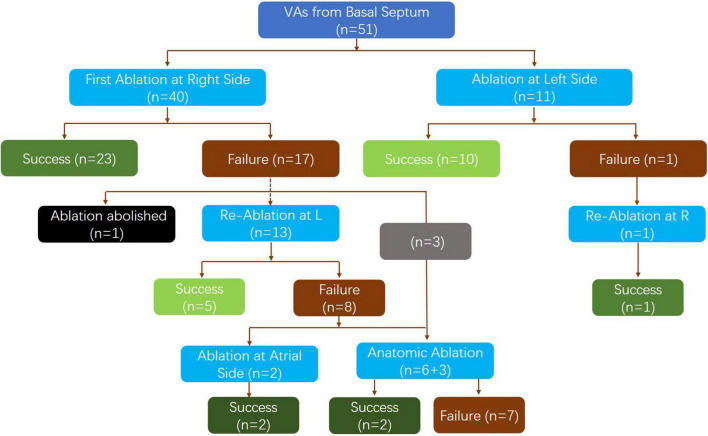
Flow chart of the detailed basal septum VA ablation results in acute term.

In the first attempt of ablation, an under-valve approach was used to target the earliest activation at the basal septum in 40 patients on the right side (Figure 1). Successful ablation of PVCs was observed in 23 patients. Remapping at the bilateral side of the basal septum was performed in the 17 patients whose initial ablation failed. At this step, the procedure was canceled in one patient due to pericardial effusion. In the remaining 16 patients, the shift of the earliest activation to the contralateral side was observed in 13 patients, while no obvious existing shift was detected in another 3 patients. Consequently, ablation at the left side was performed in 13 patients, with the VAs abolished in 5 patients. Among the 8 patients, VAs was abolished by atrial side ablation at the area of the slow pathway in two cases. Finally, anatomical ablation at both sides of the septum was performed in 9 patients, with success achieved in two patients and failure in the other 7 patients due to the transient suppression effect of VAs by ablation (4 patients) and signs of atrial ventricular conduction impairment (3 patient).

Left-side ablation was initially performed in 11 patients (retrograde aortic approach in 8 patients and transseptal approach in 3 patients due to instability of the mapping catheter in the retrograde aortic approach), and it successfully eliminated the VAs in 11 patients. After remapping on both sides, one patient who failed ablation on the left side had the VA successfully ablated by right-side ablation under the valve.

**FIGURE 2:**
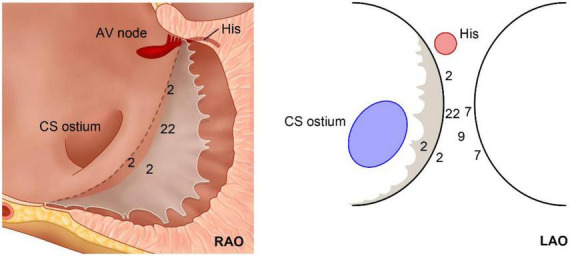
Schematic diagram of the anatomical distribution of the 51 cases of VAs.

The detailed ablation results are provided as a flowchart in Figure 1. The anatomical distribution of the VAs is delineated in Figure 2. Then, according to the site of successful ablation, patients were categorized into Group 1 (right side), Group 2 (left sight) and Group 3 (intramural foci).

### Acute and long-term outcomes

Acute procedural success was achieved in 44 patients (86.3%) in the study group and in 63 patients (95.5%) in the control group. After a median of 12 (6–36) months of follow-up, all anti-arrhythmia drugs were not used and 11 patients had a recurrence of their VAs, while a delayed cure was observed in 3 patients in the study group. There were 2 cases of recurrence in the control group (*p* = 0.002). In conclusion, success was achieved in 36 patients (70.6%) in the study group and 61 patients (90.9%) in the control group.

Totally, junctional rhythm was observed in 5 patients during ablation, with no atrial ventricular block occurred after ceasing of burning in time. First-degree atrial ventricular block was observed in 2 patients during the procedure, in which the target site was close to the right His-bundle recording site (the distance was 6.0 and 5.5 mm, respectively), and they did not recover during the follow-up. Complete atrial ventricular block was observed in one patient 3 months after the procedure, with the ablation site at mid-septum under the valve.

## Discussion

### Major findings

In this study, we described the clinical, electrophysiological and ablation results of VAs ablated from the basal septum of the ventricle. We found that (1) VAs from the basal septum may represent a different group of VAs compared to VAs from other areas of the annulus; basal septum VAs manifest unique clinical characteristics and different ablation outcomes. (2) Compared with those originating in the tricuspid annulus (TA) and mitral annulus (MA), VAs originating from the basal septum of the ventricle had more recurrence. (3) Due to the deep location and multiple exits of this group of VAs, a systematic approach is needed, including under-valve or above-valve approaches for the right side and retrograde aortic or transseptal approaches for the left side.

### Anatomical considerations and ECG recognition

The basal septum delineated in the present study could be considered a combination of the farthest left part of the tricuspid annulus and the basal inferoseptal aspect of the left ventricular wall, i.e., the posterior-superior process of the left ventricle (PSP-LV). In some recent studies, the latter was delineated as the “basal inferoseptal left ventricle” (9). Theoretically, this area could also be considered the farthest right portion of the mitral annulus; however, most studies did not delineate the VAs originating here as the “septal mitral annulus,” mostly because of the non-parallel position of the TA and MA. Based on the definition of the study, on the superior boundary of the basal septum lies the right fibrous trigone, and the inferior side was the upper end of the cardiac-crux area formed by the interventricular groove. Additionally, above this pyramidal structure lies the inferior wall of the right atrium, as well as the anteroinferior wall of the non-coronary cusp.

**FIGURE 3:**
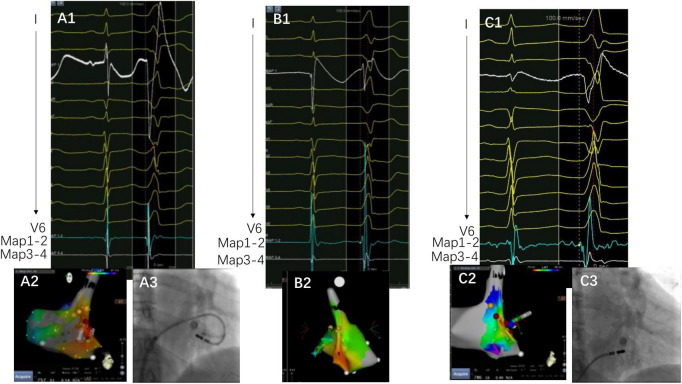
A case of VAs ablated from the low Koch’s triangle. Panels **(A1–A3)** are target activation, three-dimensional map, and fluoroscopy image at the under-valve area, respectively. The target electrogram precedes the QRS onset by 14 ms, with a small “a” signal at the target electrogram. Panels **(B1,B2)** are the earliest activation and three-dimensional map, respectively. Notice that the target electrogram has a 6 ms prematurity compared with QRS onset without the “a” potential. Panel **(C1)** is the target electrogram at the low Koch’s triangle preceding QRS onset with 28 ms. There is also an “a” potential at the target. The yellow points depict the area recording His in Panel **(C2)** and the blue point depicts the mapped activation. Notably, QRS demonstrates subtle changes after ablation on the right side of the under-valve area. Panels **(A3)** and **(C3)** depict the image contrast of the under-valve area and above valve area (low Koch’s triangle).

The complex anatomical arrangement has been translated into various VAs delineated by different groups. Hiroshi Tada et al. reported that the septum was the preferential site of origin for tricuspid annulus VAs (3). The latter has also been reported as the basal septum of the right ventricle for a distinct VA-originating structure by another group (10). Meanwhile, several studies have described the endocardial ablation of VAs from the left side, in which the structure was named “the basal infero-septal process of left ventricle” (7, 9), “left ventricular septum” (11), or “left ventricle adjacent to the membranous septum” (12). Additionally, the so-called basal infero-septal process has been successfully ablated from the atrial side (Figure 3) (13, 14).

In fact, these anatomical structures are very close to each other. Due to the neighboring topographic relationship, it is very difficult to differentiate these arrhythmias from each other before detailed mapping, and sometimes even attempted ablation. The tachycardias originating from these locations share several common ECG features, which have been shown in the present study. Almost all of the patients demonstrated an initial q or QS type wave at V1 with an abrupt reversal at V2, which has been identified as a sign of the septal accessory pathway in the ECG recognition of pre-excitation syndrome (2). Those patients eventually ablated from the right side demonstrated more QS type waves in V1; however, the QS type has little differential diagnosis value, as shown in Table 1. Nevertheless, if the ECG shows a more positive type (qR, R, RS), it is a predictive sign of warranting ablation from the left side. Additionally, the two groups demonstrated a significant difference but an obvious overlap in terms of the reversal lead.

As such, we described these tachycardias together to distinguish these arrhythmias as an entity in the present study. From a clinical point of view, it has practical significance.

### Electrophysiological findings and systematic approaches

**FIGURE 4:**
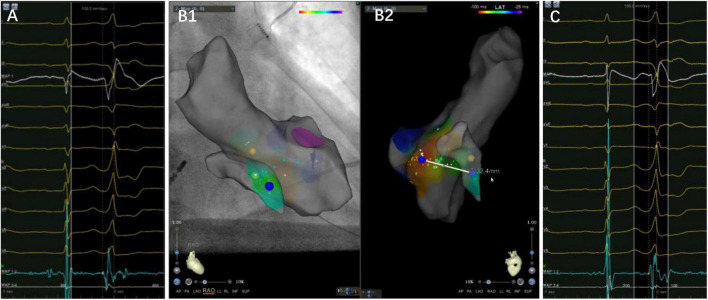
A case of premature ventricular complex ablated from the left side of the basal septum. Panels **(A–C)** are the left side target potential, three-dimensional map and the earliest activation at the under-valve area of right side, respectively. The target electrogram precedes the QRS onset by 36 ms, while the earliest activation on the right side only 14 ms, with a distance of 32.4 mm between them. Notice a small “a” potential at the target electrogram.

**FIGURE 5:**
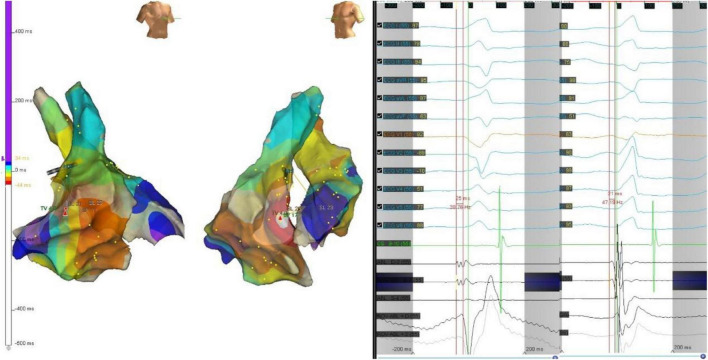
The precordial R wave transition occurred at V3 in ECG **(left)** before first ablation. Electroanatomic mapping in the right ventricle and the left ventricle, the earliest activation site was located at right basal septum with a preceding time compared with the onset of the QRS wave of 25 ms. After initial ablation failed, the precordial R wave transition occurred at V2 in ECG **(right)**. Remapping at the bilateral side of the basal septum was performed. The earliest activation site was located at left basal septum with a preceding time compared with the onset of the QRS wave of 21 ms. The PVC was successfully eliminated by ablation of both sides of basal septum.

It was found that the VAs was eliminated using the under-valve approach in 36 patients (70.6%) after a 12 month follow up. Additionally, most of the patients demonstrated a small “a” potential at the target site, which suggested that these tachycardias could be classified into a septal group of VAs from the annulus (Figure 4). Nevertheless, basal septal area VAs has unique characteristics compared with arrhythmias from other parts of the annulus due to the following facts. First, sequential ablation from both sides of the septum was required in 47% of patients due to the exit shift, which reflects a deep or even intramural location of the VA origin in some cases (Figure 5). When catheter ablation on the tricuspid valve is unsuccessful, a catheter inversion technique of the under-valve approach should be attempted for mapping and adequate contact and stability of the ablation catheter (15). In some situation, an anatomical ablation approach is also a valuable option in basal septum from intramural foci. Meanwhile, the impairment of AV conduction should not be ignored during RFCA delivered to this region. In our study, first-degree atrial ventricular block in the acute period and complete atrial ventricular block 3 months after the procedure were observed in two and one patients, respectively. Second, due to the apical displacement of the septal tricuspid valve relative to Koch’s triangle, some VAs from the septal tricuspid annulus were successfully ablated from the low Koch’s triangle with no complications (14). Third, in those patients that need left-side ablation, a combined strategy employing a retrograde aortic approach and a transseptal approach should be considered. Consequently, based on the unique anatomical and electrophysiological characteristics, a systematic approach for VAs originating from the basal septal area is warranted.

### Limitations

First, this was a retrospective, single-center study. Second, the patients in the study group were found to be older than those in the control group, as well as more comorbidities including hypertension and coronary artery disease were found in basal septum VAs. It could not be determined whether the older age and more comorbidities accounted for the relative low success rate in the study group. To clarify this issue, systematical MRI imaging is needed to illuminate if a high progression of myocardial fibrosis at the basal septum due to the age and comorbidity occurred in the study group.

## Conclusion

Based on the unique anatomical and electrophysiological characteristics of the basal septum, a systematic approach for VAs originating from this area is warranted. Moreover, the follow-up data seemed to show a relative high recurrence rate for basal septal VAs during a period of time.

## Data availability statement

The original contributions presented in this study are included in the article/supplementary material, further inquiries can be directed to the corresponding author.

## Ethics statement

The studies involving human participants were reviewed and approved by the Institutional Ethical Committee of the First Affiliated Hospital of Nanjing Medical University. The patients/participants provided their written informed consent to participate in this study.

## Author contributions

LS and CW performed the clinical study and drafted the original manuscript. HC, GY, KG, ML, MCu, HL, ZW, and MCe interpreted and discussed the data. WJ reviewed and edited the manuscript. All authors contributed to the whole article and approved the final version of this manuscript.
